# Case of Amputation of the Shoulder-Joint

**Published:** 1833-10

**Authors:** Bransby B. Cooper


*To the Editor of the Medical Quarterly Review.*

Sir: I beg leave to forward for insertion in your publication the following case, not because it led to the necessity of amputation at the shoulder-joint, (as that operation is too frequent to be a matter of professional interest,) but in consequence of the complete laceration of the axillary artery being unattended with bleeding; presenting a beautiful illustration of the process which nature employs in spontaneously suppressing hemorrhage.

I have the honour to be, sir, your very obedient servant,

Bransby B. Cooper.

CASE.

James Clark, æt. thirty-four, by employment a waggoner, was admitted into Guy's Hospital, August 29th, 1833, with a compound comminuted fracture of the left humerus, near the shoulder-joint.

He states that the accident occurred from his attempting to lock the wheel of his waggon, containing about four tons of paper, previously to his descending Shooter's hill; and this he did without first stopping his horses, so that the hinder wheel knocked him down, and passed over his arm. The patient says that he lost a considerable quantity of blood upon the spot where the accident happened; and when brought to the hospital, about an hour and a half afterwards, his pale countenance, and small jerking pulse, indicated that such had been the case.

Upon examination, I found a wound upon the inner side of the upper part of the arm, just above the insertion of the pectoralis major muscle; and, upon putting my finger into this large lacerated opening, I discovered that the humerus was not only fractured, but severely comminuted, and the upper portion of the fractured bone so near to the wound as to leave no doubt that it had occasioned the laceration of soft parts, which were also extensively contused. The extreme phalanges of the fore, middle, and ring fingers were completely smashed; and the patient had lost all sensation and motion in the arm, below the fracture.

Upon exploring the depth of the wound with the finger, it was ascertained that it sunk deeply into the axilla, where the pulsation of the axillary artery could be felt, beating just as if it had been compressed by a ligature; but immediately below this point no vestige of artery could be detected; neither was there any sensation of pulsation to be discovered below the upper part of the wound. The axillary plexus was also partially torn through. When I first began to examine this patient, and until indeed I discovered that the axillary artery and nerves were lacerated, I intended to have attempted the preservation of the limb; but, upon considering the injury sustained by the surrounding soft parts, and the laceration of the blood-vessels and nerves, I felt assured that I was right to remove at once by amputation what would otherwise probably have proved the cause of death. I therefore pointed out to him the propriety of his submitting to the operation, as the only hope of saving his life, there being no chance left of preserving the limb. He readily consented, and, being carefully conveyed into the operating theatre, I removed his arm at the shoulder-joint, in the following manner:

Having placed him on a low chair, with a folded sheet around his body, held by an assistant, for the purpose of giving the patient support, I raised his injured arm, so as to separate it from its side to the angle, which I thought most convenient for the completion of the operation; and then, and not till then, I requested Mr. Callaway to compress the subclavian artery above the clavicle. I speak of the precise moment at which the vessel was compressed, because, as has been before observed by authors, there should be no further occasion for moving the limb after the subclavian has been pressed upon the first rib, as the slightest change of position may liberate the vessel from the assistant's command. With a very strong and large scalpel, I then commenced an incision from about the centre of the spine of the scapula, and continued it downwards to the insertion of the deltoid muscle; cutting deeply, so as to divide the muscles in one sweep, down to the bone. I commenced my second incision at about the junction of the middle with the inner third of the clavicle, and continued it downwards to meet the termination of my first: this cut was not so deep, as, from the laceration of the skin and part of the pectoralis major, I had much less substance to cut through. The deltoid was now detached from the humerus and capsular ligament, and turned up, attached at its base by its origin, so as to form a triangular flap. The capsular ligament was now opened, and the head of the humerus readily disarticulated, when, instead of removing the whole arm at once, as is usually done, I detached the upper portion of the bone which was completely separated from the remainder by the fracture.

Thus far in the operation there was little or no bleeding, although Mr. Callaway did not press upon the subclavian artery, finding there was no occasion for it. The operation was now completed by cutting through the remaining soft parts which held the limb attached to the trunk.

The axillary artery, with several of its branches, could now be seen plugged up with coagulated blood, so as completely to account for the absence of hemorrhage. From the extremities of these vessels portions of coagulated blood could be seen extending from their open mouths, from an inch and a half to two inches in length, and apparently to a considerable extent within their calibre, as indicated by their distention, colour, and feel. This coagulum, until the period of that profound physician, Dr. Jones, whose book on hemorrhage should be in the library of every surgeon, was considered as the sole means of suppressing hemorrhage; but he has shown that, however much the coagulum may assist in staying loss of blood, during the diminished action of the heart under approaching syncope, still it is rather to be considered as essential in effecting this great object, by rapidly inducing an adhesive inflammation in the coats of the lacerated artery and surrounding cellular membrane, and thus permanently sealing the open mouth of the vessel, than as checking the bleeding merely as a mechanical agent. It is this coagulable lymph which fills up the interstices between the coats of the artery, and consolidates the surrounding cellular membrane, so as to form that solid cord, the remains of an obliterated artery. Firmly as the coagulum was fixed in the axillary artery, I did not think it right to run the risk of trusting to this process of suppressing hemorrhage, and therefore placed a ligature around the main trunk, and one or two of its important branches. Having removed the fibro-cartilaginous rim of the glenoid cavity, I secured the upper to the lower flap by three sutures and adhesive plaster. The patient was then put to bed, and ordered immediately to take forty drops of laudanum in an ounce and a half of camphor mixture.

30th. The patient slept very well; bowels open once; tongue slightly coated, but moist; no pain; pulse 100, and rather full.

31st. Slept well; bowels open twice; tongue moist, and somewhat coated. There is considerable oozing of bloody serum from the wound.

September 1st. Did not sleep quite so well; complains of some pain in the neck just above the posterior part of the wound, where however there does not appear to be any puffiness. The wound was dressed this morning, and a considerable quantity of bloody serum was discharged: it seems, indeed, as if there were a considerable coagulum of blood at the anterior and superior edge of the flap, the inferior part of which was so much contused at the time of the injury as now to be in a sloughy state: the rest of the wound, however, looks very well. Tongue moist; bowels open; skin natural; pulse 106, less full. Ordered some beef-tea. ℞. Sulphat. Quin. gr. ij.; Liq. Opii Sed. m. iv.; Inf. Rosæ comp. ℥iss. M. fiat haustus, 4ta quâque hora sumend.

Four P.M. The pain in the neck is rather increased; pulse 120, slightly jarring; skin moist; tongue rather dry; is somewhat restless. ℞. Liq. Opii Sed. gtt. xv. statim sumend.

2d. Slept tolerably well, talking a little however in his sleep; pain in his neck considerably diminished; bowels open; skin rather hot, but bedewed with perspiration; tongue coated, but moist, except in the centre; pulse 112, still rather jarring.

Nine P.M. Is much easier; has now little or no pain in the neck; pulse 110, and rather less jarring; tongue coated, but moist. Has slept a little this evening.

3d. Eight A.M. Did not sleep well last night; the pain in the neck however, has entirely subsided; pulse 125, irritable and jarring; skin moist; tongue coated, and moist; bowels not open this morning: complains of some pain in his right leg and thigh. Wound looks very unhealthy, and somewhat emphysematous in parts; has no pain in or about the flaps. Ordered some porter, and to continue the beef-tea, &c.

Half-past three P.M. The pain in the leg is considerably increased; countenance rather anxious; respiration quicker than natural; pulse 136, jarring and irritable; skin a little moist; tongue rather dry, and slightly brown in the centre. Feet rather cold: to have warm-water bottles applied to them. Complains of some pain and cramp in the arm. ℞. Tr. Opii, m. xxx.; Julep. Ammon. ℥iss. M. fiat haust. statim sumendus.

Five P.M. Has less pain in his leg, but it is much increased in the arm; bowels open; tongue dry; skin slightly moist; pulse 130, weak and jarring; some delirium. To continue the beef-tea.

Nine P.M. Less pain and cramp; the delirium continues; pulse 132; tongue coated and dry; skin moist; feet warm; countenance anxious; great restlessness; some twitchings of the right arm, in which, as well as in the leg of the same side, he complains of pain, especially in the joints. Wound looks very sloughy, and the emphysematous appearance has extended considerably, even under the pectoralis major. ℞. Morphiæ muriat. gr. i. statim, et gr. ss. rep. omni horâ si opus sit.

The patient, after this, took two half-grain doses of the muriate of morphia; notwithstanding which, the pain in the arm and leg, as also the twitchings, increased, and the delirium became complete raving. At

Twelve P.M. the spasm and pain in the right leg and arm became aggravated to an alarming extent, the raving continued, and the face was drawn somewhat to one side. In this state he continued for about twenty minutes, when he suddenly fell back, and expired.

His body was examined twenty-four hours after death, when the whole wound presented so sloughy an appearance, that one structure could scarcely be distinguished from another.

On opening the chest, the pleura upon the left side was found very much inflamed, and there were recent adhesions of the pleura pulmonalis to the pleura costalis. The brain indicated no morbid changes, although, from the symptoms immediately prior to his death, (there being a tendency to opisthotonos,) it was clear that he died under a high degree of nervous excitement, amounting nearly to tetanus.

On examining the artery, it was found perfectly sealed by the adhesive process.

